# A ten-year numerical hindcast of hydrodynamics and sediment dynamics in the Loire Estuary

**DOI:** 10.1038/s41597-023-02294-w

**Published:** 2023-06-22

**Authors:** Florent Grasso, Matthieu Caillaud

**Affiliations:** Ifremer – DYNECO/DHYSED, Centre de Bretagne, CS 10070, Plouzané, F-29280 France

**Keywords:** Physical oceanography, Environmental impact

## Abstract

A numerical hindcast of the macrotidal Loire Estuary (France) has been generated to provide a long-term dataset (2008–2018) of estuarine hydrodynamics, temperature, salinity, and sediment dynamics. This hindcast is based on simulations coupling water motion, wave and mixed sediment models, forced with realistic conditions and extensively validated in the salinity gradient and turbidity maximum areas. These data represent extremely valuable information for diverse scientific communities, providing (i) environmental parameters for ecosystemic studies along the Loire River–sea continuum, (ii) a singular estuarine configuration for inter-comparison of estuarine functioning, and (iii) a ten-year synoptical view of a major estuarine environment of the North Atlantic Ocean.

## Background & Summary

Estuaries represent crucial interfaces along the land-sea continuum, impacted by marine, riverine, and atmospheric forcing. The mixing between freshwater and seawater strongly impacts the fate of dissolved and particulate matters between continental and marine environments (e.g., nutrients, contaminants and sediments)^1^. Moreover, estuarine circulation and tidal currents can induce high levels of suspended sediment concentration (SSC) and form estuarine turbidity maxima (ETM)^2–12^, directly affecting estuarine morphodynamics and biogeochemical processes^13^. In addition to natural forcing, human-induced changes (e.g., estuary deepening and narrowing) have drastic impacts on the physical and ecological estuary functioning^14–18^. Exceptional hydro-meteorological conditions, associated with inter-annual variability and/or climate changes, exacerbate drought, flood and stormy periods that have a major impact on estuary physics. Therefore, it is essential to understand the estuarine dynamics during such critical periods (from events to years) to be able to provide insights into estuary trajectories under future climatic and human pressures^15^.

Recent developments in realistic numerical models, i.e., based on realistic bathymetry and forcing conditions, enable hindcasts to be generated over decadal periods^15^. Such multi-year simulations, validated with long-term *in-situ* high-frequency monitoring networks^9,19,20^, provide valuable four-dimensional parameters (i.e., horizontal, vertical, and temporal components) of environmental conditions, such as water level and current, temperature, salinity, SSC, and bed substrate composition. Such hindcasts are especially useful: (i) to study the impacts of anthropogenic and climate changes on estuarine physics and ETM dynamics;^11,15,16,21–23^ and (ii) to provide abiotic explanatory parameters for biogeochemical and ecological studies^24–27^.

**Fig. 1:**
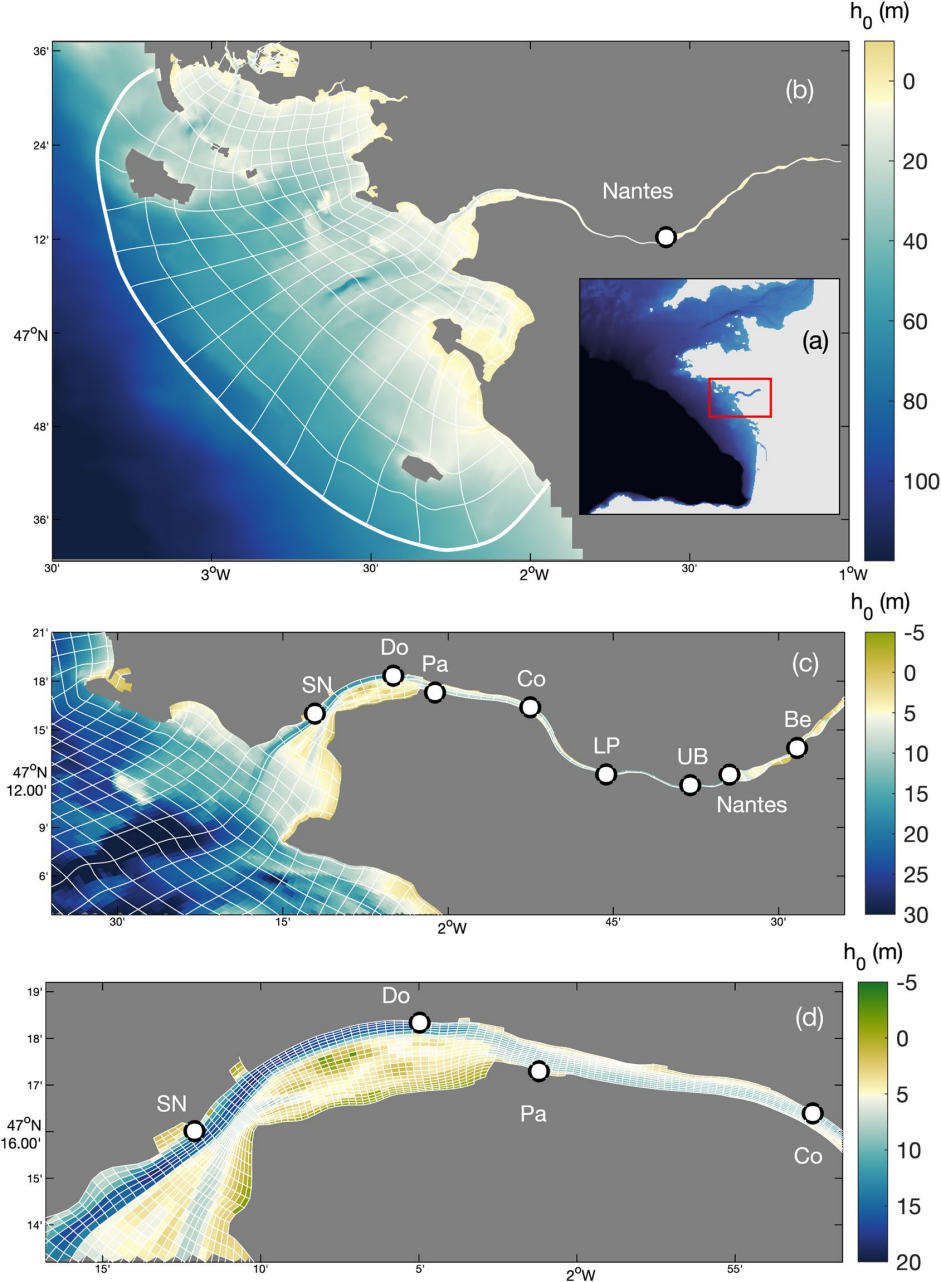
Bathymetry of the Loire Estuary, France (with h_0_ the water depth relative to mean sea level). (**a**) Location of the estuary in the Bay of Biscay (Western French Atlantic Coast), (**b**) full domain of the MARS3D model with every tenth grid cell represented, (**c**) focus on the estuary with every fifth grid cell represented, and (**d**) focus on the lower estuary with every grid cell represented. Black circles represent locations of water level, salinity, and SSC comparisons: Saint Nazaire ‘SN’, Donges ‘Do’, Paimboeuf ‘Pa’, Cordemais ‘Co’, Le Pellerin ‘LP’, Usine Brulée ‘UB’, and Bellevue ‘Be’.

**Fig. 2:**
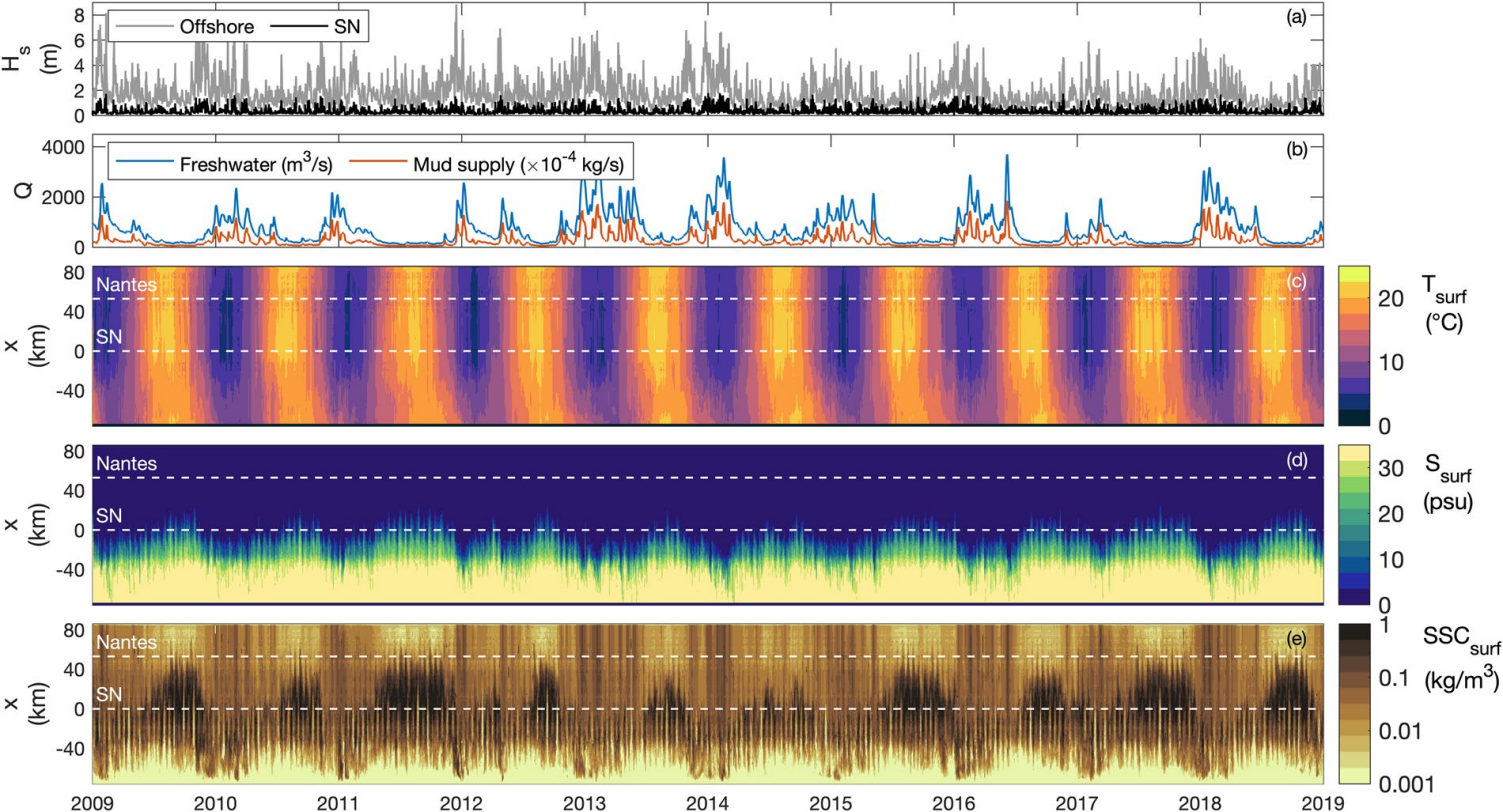
Numerical hindcast of the Loire Estuary (2009–2018). (**a**) Significant wave height H_s_ at the offshore model boundary (grey) and at Saint Nazaire ‘SN’ (black), and (**b**) Loire River discharge Q at the upstream model boundary for freshwater (blue) and mud supply (orange). Width-averaged along-estuary transects of surface: (**c**) temperature T_surf_, (**d**) salinity S_surf_, and (**e**) suspended sediment concentration SSC_surf_. In (**c**–**e**), white-dashed lines represent Saint Nazaire ‘SN’ and Nantes locations, at x = 0 and 53 km respectively.

Numerical hindcasts have recently been realised for two of the three largest French estuaries (Seine^15^ and Gironde^21^), arousing interdisciplinary studies and tackling challenges on habitats and species distributions^24–26^. The aim of this study is to complete these datasets with a ten-year hindcast (2008–2018) for the Loire Estuary (France; Figs. 1, 2). On the one hand, it offers environmental parameters for ecosystemic studies along the Loire River–sea continuum; and on the other hand, it provides a contrasted estuarine configuration for inter-estuary comparisons^5,28–32^.

The Loire Estuary is the second largest French estuary, extending 100 km from Saint Florent-le-Vieil at the upstream limit of tidal influence to the Bay of Biscay on the Atlantic Coast (Fig. 1a,b). The estuary has a semi-diurnal macrotidal regime with a tidal range varying from 1.5 to 6.4 m at Saint Nazaire close to the estuary mouth (Fig. 1c). Tides and river flow induce large seasonal, fortnightly, and semi-diurnal salinity changes^27^. Moreover, the Loire Estuary is characterized by a well-developed turbidity maximum (i.e., SSC exceeding 0.5 g/l at the surface and ETM mass up to 1 Mt)^9^, migrating from Nantes to Donges during low to high river discharges (Fig. 1c,d). Between 2009 and 2018, the Loire River discharge ranged from 100 to 3700 m^3^/s, with a mean freshwater runoff of around 730 m^3^/s and a terrigenous sediment load of around 1.1 Mt/year.

## Methods

A realistic three-dimensional numerical model has been set up to simulate the hydrodynamics, temperature, salinity, and sediment dynamics along the Loire Estuary. Similar models have previously been implemented in the Seine^11,15,16,22,23,25,33^ and Gironde^21,24,26^ estuaries (France). Hereafter is presented an overview of the model characteristics both in terms of hydrodynamics and sediment transport.

### Hydrodynamic model

The model is based on a non-nested (i.e., unique) configuration using the hydrostatic model MARS3D^34^. An orthogonal curvilinear grid is used to better represent the estuarine shape and to optimize computational costs while refining the grid resolution in some specific areas (i.e., in the river meanders, in the central estuary, and at the estuarine mouth; Fig. 1). Horizontal cell size ranges from around 50 m in the meanders to approximately 1.3 km offshore while the vertical grid is divided into 10 equidistant sigma layers.

The 114 main tidal components, extracted from the CST France database, https://maree.shom.fr (Service Hydrographique et Océanographique de la Marine, SHOM), are used to force the circulation at the open boundaries. Surges, provided by a configuration of the two-dimensional MARS2D model applied to a larger domain (i.e., over the Bay of Biscay), are added to the water elevation at these same boundaries. Realistic freshwater discharges and sediment loads are prescribed at the upstream boundary of the Loire River (i.e., Saint Florent-le-Vieil) and at the Vilaine Estuary mouth for the Vilaine River (see further details on river supplies in the “Data records” section). In addition, the model is forced by wind stresses and pressure gradients obtained from the high‐resolution meteorological AROME model (Météo‐France): https://donneespubliques.meteofrance.fr. The simulated turbulence is based on a *k*-ε turbulence closure scheme. Waves are simulated with the WAVEWATCH III® (WW3) numerical model^35^ using the same computational grid as the one used by MARS3D in this study. The hourly free surface elevation and current velocity provided by the MARS3D hydrodynamic model, along with local winds and swell data extracted from a larger model, were used to force the WW3 configuration. However, the wave effects on hydrodynamic circulation are not taken into account because of the dominance of tidal currents over wave-induced currents. The bottom orbital velocities simulated by the wave model were used to compute the wave-induced bed shear stress. Finally, the total bed shear stress (τ) was expressed as a combination of the current-induced and wave-induced bed shear stresses, accounting for non-linear interactions following the formulation of Soulsby^36^.

The hydrodynamic bottom roughness *z*_*0b*_ is spatially distributed according to the observed sediment substrate. However, the sediment nature in the estuary changes according to the ETM location and the presence of fluid mud layers, which depend on the Loire River discharge. Therefore, following an approach adopted by ARTELIA^37^, the bottom roughness also depends on the Loire River discharge^38^. More details on the hydrodynamic model configuration are provided by Khojasteh Pour Fard^39^.

### Sediment transport model

The hydrodynamic model is coupled with the process-based, multiclass, multilayer sediment transport model MUSTANG^40–42^, which computes the temporal and spatial variations of sand and mud content in the bed under hydrodynamic forces and consolidation process. The MARS3D-MUSTANG coupling resolves advection-diffusion equations in the water column and sediment exchanges between the bed and the water column for different particle classes. Based on hundreds of granulometric samples collected in the Loire Estuary^43^, five sediment classes are prescribed in this hindcast: one mud, three sands, and one gravel (see sediment class diameters in Table 1). Sediment classes are initially distributed according to bed substrate observations from the SHOM.

**Table 1:** Main sediment model calibration parameters.

Particle diameter		*Gravel*	1 mm	*Based on local granulometric data*
		*Medium sand*	450 µm	
		*Fine sand*	200 µm	
		*Very fine sand*	100 µm	
		*Mud*	<63 µm	
Mud settling velocity (Eq. 1)		*w*_*s,min*_	0.1 mm/s	
		*w*_*s,max*_	1 mm/s	
		*c*_1_	0.003	
		*c*_2_	0.79	
		*a*	0.3	
		*b*	0.18	
Erosion law	Non-cohesive	*n*_*sand*_	1.6	
	Cohesive	*n*_*mud*_	1	
		*E*_*0,mud*_	10^−3^	
		*α*_*1*_	10^−5^	
		*α*_*2*_	2	

Non-cohesive sediment classes (sands and gravel) have constant settling velocities depending on their diameters^36^. The coarser classes are transported in the bottom layer only, except for the very fine sand, which was treated in three dimensions. In two dimensions, the velocity in the bottom layer is corrected to account for a logarithmic profile for the velocity in the whole water column, and the calculated sand concentration is then assumed to follow a Rouse profile^44^. The mud class is computed as a three-dimensional variable with a settling velocity *w*_*s,mud*_ varying with concentration and turbulence to represent flocculation processes following van Leussen^45^:

1 $${w}_{s,mud0}={\rm{\min }}\left[{w}_{s,max},{\rm{\max }}\left({w}_{s,min},{c}_{1}.{C}_{mud}^{{c}_{2}}.\frac{1+aG}{1+b{G}^{2}}\right)\right]$$

with *C*_*mud*_ the mud concentration (kg/m^3^), *G* the turbulent shear rate (s^−1^), and *w*_*s,min*_, *w*_*s,max*_, *a*, *b*, *c*_1,_ *c*_*2*_ calibration parameters detailed in Table 1. A dependency between the mud settling velocity and salinity (*S*) is also considered to account for the influence of salinity on flocculation^46^: below a critical salinity of 5 psu, the mud settling velocity decreases with salinity (see details in Diaz, *et al*.^21^).

The erosion flux is based on Partheniades-Arathurai equation^47^:

2 $$\left\{\begin{array}{c}\tau  > {\tau }_{ce}\Rightarrow E={E}_{0}{\left(\frac{\tau }{{\tau }_{ce}}-1\right)}^{n}\\ \tau  < {\tau }_{ce}\Rightarrow E=0\end{array}\right.$$

with *E* the erosion flux, *E*_*0*_ an erodibility parameter (kg/m^2^/s), *τ*_*ce*_ the critical shear stress for erosion (N/m^2^), and *n* a calibration parameter. A distinction between cohesive and non-cohesive sediment behaviours are made based on the mud fraction in the bed surficial layer (*f*_*m*_). In both cases, the Partheniades equation was prescribed with different calibration parameters. For a non-cohesive behaviour (*f*_*m*_ < *f*_*m,cr*1_ where *f*_*m,cr1*_ = 1000.*d*_*50,sand*_, with *d*_*50,sand*_ the weighted mean diameter of sand classes in the surficial layer), the erosion regime follows a pure sand behaviour. The critical shear stress for erosion is determined by the Shields criteria^36^, the erosion rate is derived from erodibility measurements^48^, and the calibration parameter *n* is defined as *n*_*sand*_ (Table 1). In the presence of a cohesive seabed (*f*_*m*_ > 0.7; Le Hir, *et al*.^41^), the formulation follows a pure mud erosion regime with *n* = *n*_*mud*_ and *E* = *E*_*0,mud*_ (Table 1). The critical shear stress for mud erosion *τ*_*ce,mud*_ depends on the bed consolidation state, which is represented by the relative mud concentration (*C*_*relmud*_) through a classical power law *τ*_*ce,mud*_ = *α*_*1*_.*C*_*relmud*_^*α*2^ (see Grasso, *et al*.^40^), with *α*_1_ and *α*_2_ defined in Table 1. Here, *C*_*relmud*_ is defined as the mud concentration in the space between sand particles^49^. Finally, for a mixed erosion regime, the erosion law parameters are linearly interpolated between pure sand and pure mud behaviours. The main empirical parameters are identified in Table 1 and further details on the formulations used in this model can be found in Grasso, *et al*.^11^ and Diaz, *et al*.^21^.

The deposition flux is calculated using a critical shear stress for deposition for each sediment class following the law of Krone^11,21,41,50^. Sediment sliding along the slope is taken into account to prevent an excessive increase of bed slope between depositing banks and the eroding channel. This process is computed by assigning a part of the deposition flux from one cell to the neighbouring one based on the slope between the two cells. The fraction of fresh deposit transposed to a deeper adjacent cell linearly depends on the local slope.

Hindcast simulations over the 2008–2018 period were run through independent years following a morphostatic approach, i.e., no morphodynamic coupling, which is relevant when morphological changes remain relatively small to hydrodynamic processes^15^. This assumption holds for the 2008–2018 period because the Loire Estuary is heavily dredged at a constant depth and no significant changes have been observed in the bathymetry since 2000^9,29^. This is confirmed by the model validation presenting similar skills in 2008 and 2018 (Table 2). Each year was run twice to consider a 1-year spin-up period before analysing the half-hourly outputs^11,16,23^.

**Table 2:** Numerical model skills (i.e., correlation coefficient r^2^, root mean square error e_rms_, and bias b) for water level, salinity and SSC at Bellevue ‘BE’, Usine Brulée ‘UB’, Le Pellerin ‘LP’, Cordemais ‘Co’, Paimboeuf ‘Pa’, Donges ‘Do’, and Saint Nazaire ‘SN’.

Stations			SN	Do	Pa	Co	LP	UB	Be
Water level	*ζ*	*r*^2^	**0.99** 0.99	**0.98** 0.99		**0.97** 0.97	**0.97** 0.98	**0.96** 0.97	
		*e*_*rms*_ (m)	**0.15** 0.14	**0.20** 0.16		**0.26** 0.25	**0.23** 0.22	**0.27** 0.24	
		*b* (m)	**−0.02** 0.00	**0.00** **−**0.06		**0.02** 0.03	**−0.04** **−**0.05	**−0.01** **−**0.04	
Surface salinity	*Sal*_*hf*_	*r*^2^			**0.91** 0.79		**0.69** 0.65		
		*e*_*rms*_ (psu)			**2.6** 3.5		**0.5** 0.5		
		*b* (psu)			**−1.7** 0.7		**−0.3** **−**0.1		
	*Sal*_*tide*_	*r*^2^			**0.96** 0.91		**0.73** 0.78		
		*e*_*rms*_ (psu)			**1.9** 1.9		**0.4** 0.3		
		*b* (psu)			**−1.6** 0.6		**−0.3** **−**0.1		
Surface suspended sediment concentration	*SSC*_*hf*_	*r*^2^			**0.12** 0.09	**0.14** 0.42	**0.29** 0.3		**0.35** 0.38
		*e*_*rms*_ (kg/m^3^)			**1.10** 0.49	**1.07** 0.56	**0.59** 0.75		**0.03** 0.008
		*b* (kg/m^3^)			**−0.38** 0.00	**−0.41** **−**0.12	**−0.21** **−**0.30		**0.01** **−**0.01
	*SSC*_*tide*_	*r*^2^			**0.40** 0.19	**0.51** 0.87	**0.71** 0.65		**0.42** 0.41
		*e*_*rms*_ (kg/m^3^)			**0.61** 0.31	**0.77** 0.32	**0.47** 0.59		**0.03** 0.05
		*b* (kg/m^3^)			**−0.40** **−**0.02	**−0.42** **−**0.12	**−0.21** **−**0.31		**0.01** **−**0.01

Bold and normal font type values correspond to the 2008 and 2018 simulations, respectively. ‘hf’ and ‘tide’ subscripts define high-frequency (i.e., every 30 minutes) and tide-averaged values, respectively.

## Data Records

### Hindcast repository

The data files containing the results of the Loire Estuary hindcast, i.e., hydrodynamics, temperature, salinity, and sediment dynamics, are available on the CurviLoire Hindcast repository^51^.

The repository is structured in four directories:

- /hydro (NetCDF files): 3D half-hourly results for water column variables:
  - Hydrodynamics: water depth ‘*H0*’, water level ‘*XE*’, horizontal current velocity ‘*U,V*’;
  - Temperature ‘*TEMP*’, salinity ‘*SAL*’;
  - Waves: significant height ‘*hs*’, direction ‘*dir*’, bottom orbital velocity ‘*ubrx,ubry*’;
  - Suspended sediment concentration: mud ‘*Mud*’, very fine sand ‘*Veryfinesand*’, fine sand ‘*Finesand*’, medium sand ‘*Mediumsand*’, and gravel ‘*Gravel*’.
- /sedim (NetCDF files): 3D half-hourly results for bed compartment variables:
  - Bed shear stress: wave-induced ‘*TENFONW*’, current-induced ‘*TENFONC*’, and total components ‘*TENFON*’;
  - Sediment bed: total thickness ‘*EPTOT*’, number of layers ‘*NBNIV*’, layer thickness ‘*DZS*’;
  - Bed sediment concentration: mud ‘*Mud*’, very fine sand ‘*Veryfinesand*’, fine sand ‘*Finesand*’, medium sand ‘*Mediumsand*’, and gravel ‘*Gravel*’.
- /source_code (fortran files): the MARS3D-MUSTANG source codes used to provide the hindcast;
- /param_files (ascii files): the parameter files used to configure the simulations.

### Hindcast forcing conditions

The hindcast gathers eleven individual years of simulations (from 2008 to 2018) with the following forcing conditions:

- Bathymetry: 2008;
- Atmospheric forcing: Météo-France AROME (1.3 × 1.3 km² spatial resolution and hourly outputs);
- Open boundary conditions:
  - Tides: 114 main tidal components extracted from the CST France database (SHOM);
  - Storm surges: MARS2D-MANGAE2500 (2.5 × 2.5 km^2^ spatial resolution and hourly outputs);
  - Waves: WAVEWATCH III®-NORGASUG 4 (5 × 5 km^2^ spatial resolution and hourly outputs).
- River discharge:
  - Freshwater: daily-measured runoffs of the Loire and Vilaine rivers (*Q*_*L*_ and *Q*_*V*_, respectively);
  - Sediment load: suspended mud concentration (*SMC*, in g/l) associated with the river runoff following the following relationships:
    - Vilaine^52^: $$SM{C}_{V}=\left(0.031\ast {Q}_{V}+17\right)/1000$$
    - Loire^37^: $$\left\{\begin{array}{c}{\rm{Normal}}\;{\rm{conditions}}:SM{C}_{L}={\rm{\max }}\left(5,0.036\ast {Q}_{L}\right)/1000\\ {\rm{Flood}}\;{\rm{increase}}:SM{C}_{L}={\rm{\max }}\left(5,0.05\ast {Q}_{L}\right)/1000\\ {\rm{Flood}}\;\mathrm{decrease}:SM{C}_{L}={\rm{\max }}\left(5,0.023\ast {Q}_{L}\right)/1000\end{array}\right.$$

## Technical Validation

The numerical hindcast has been validated with the water level, salinity and SSC measurements in 2008 and 2018 provided by the GIP Loire Estuaire in the framework of the SYVEL high-frequency continuous monitoring network: https://www.loire-estuaire.org/accueil/nos-outils/reseau-de-mesures-en-continu-syvel-2. Simulations are compared after a one-year spin-up period.

### Water level

**Fig. 3:**
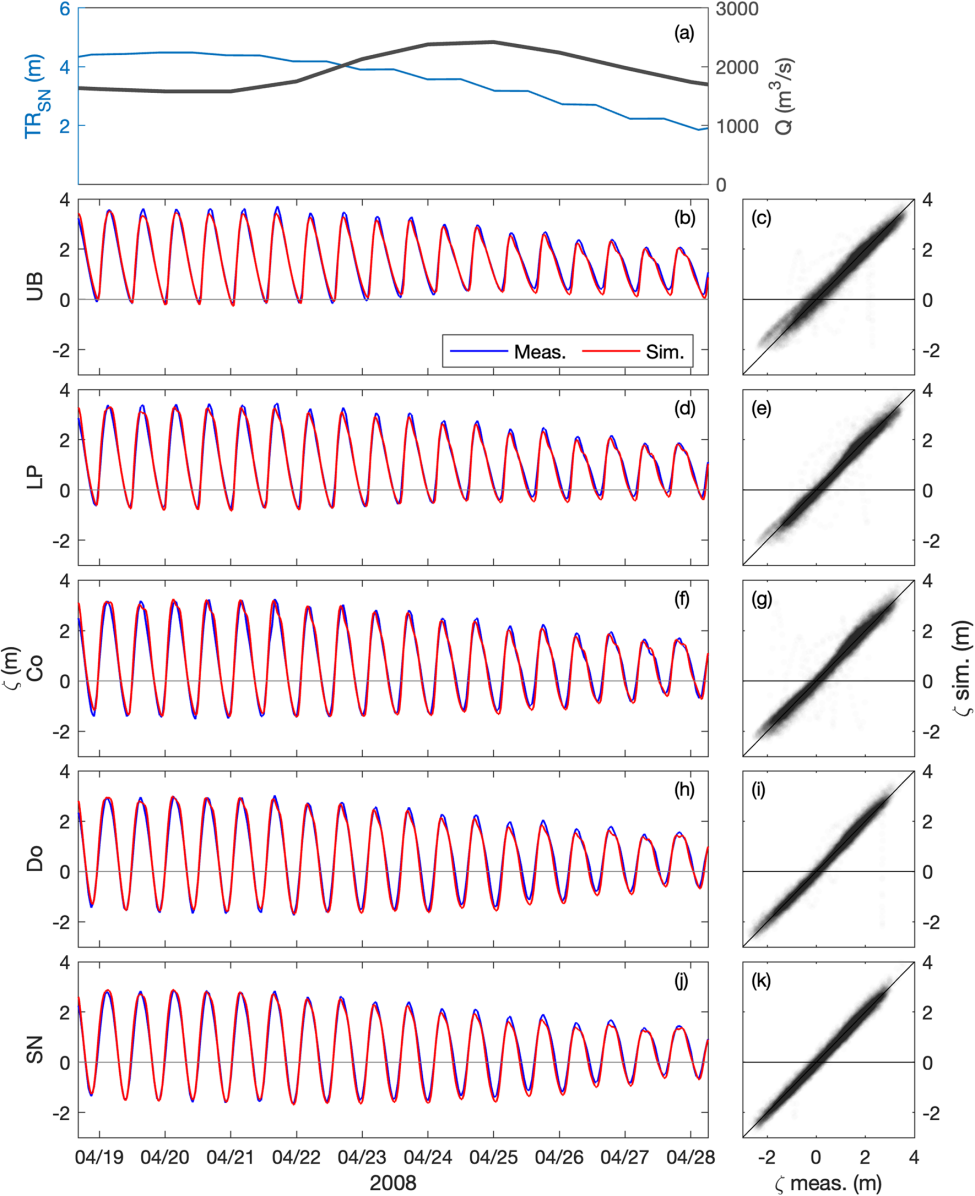
Hindcast validation of water levels *ζ* along the Loire Estuary. (**a**) Loire River discharge *Q* at the upstream model boundary and tidal range *TR*_*SN*_ at Saint Nazaire ‘SN’ (blue). Water surface elevations at (**b,c**) Usine Brulée ‘UB’, (d-e) Le Pellerin ‘LP’, (**f,g**) Cordemais ‘Co’, (**h,i**) Donges ‘Do’, and (**j,k**) Saint Nazaire ‘SN’ (see station locations in Fig. 1c). (Left panels) Measurements (blue) and simulations (red) from April 19 to 28, 2008. (Right panels) simulations versus measurements from January to December 2008.

**Fig. 4:**
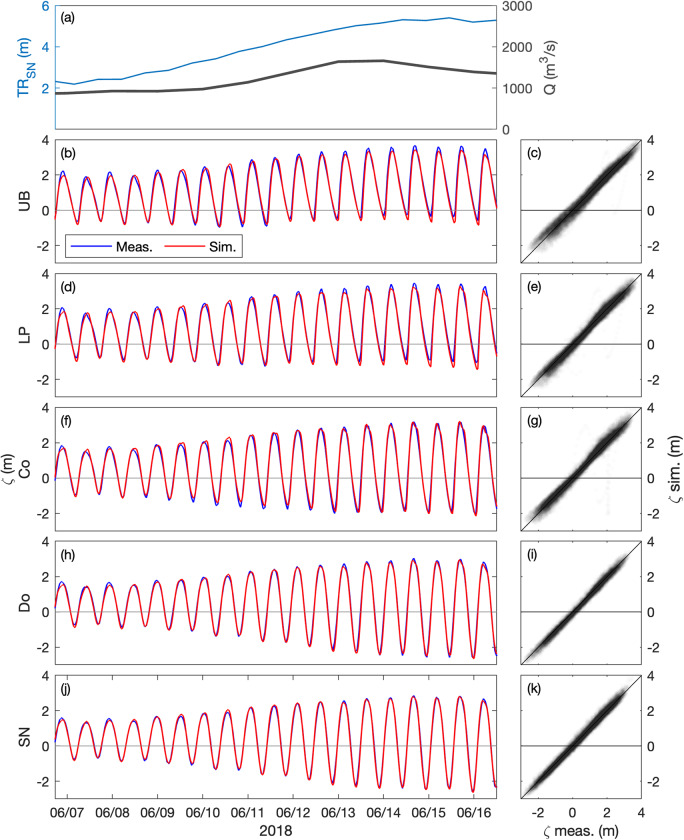
Hindcast validation of water levels *ζ* along the Loire Estuary. (**a**) Loire River discharge *Q* at the upstream model boundary and tidal range *TR*_*SN*_ at Saint Nazaire ‘SN’ (blue). Water surface elevations at (**b,c**) Usine Brulée ‘UB’, (**d,e**) Le Pellerin ‘LP’, (**f,g**) Cordemais ‘Co’, (**h,i**) Donges ‘Do’, and (**j,k**) Saint Nazaire ‘SN’ (see station locations in Fig. 1c). (Left panels) Measurements (blue) and simulations (red) from June 7 to 16, 2018. (Right panels) simulations versus measurements from January to December 2018.

Free surface elevation is compared at five tidal gauges along the estuary, where the salt wedge and turbidity maximum take place (i.e., Saint Nazaire ‘SN’, Donges ‘Do’, Cordemais ‘Co’, Le Pellerin ‘LP’, and Usine Brulée ‘UB’; Fig. 1c). Measured and simulated water levels are presented over a spring-neap tidal cycle in April 2008 and June 2018, illustrating the tidal propagation from Saint Nazaire to Usine Brulée (left panels in Figs. 3, 4). The model properly simulates the tidal variations and correctly captures the tidal asymmetries along the estuary. Comparisons over the entire years (right panels in Figs. 3, 4) provide good agreements with correlation coefficients *r*^2^ ≤ 0.96 and root mean square errors *e*_*rms*_ ≤ 0.27 m (Table 2).

### Salinity

**Fig. 5:**
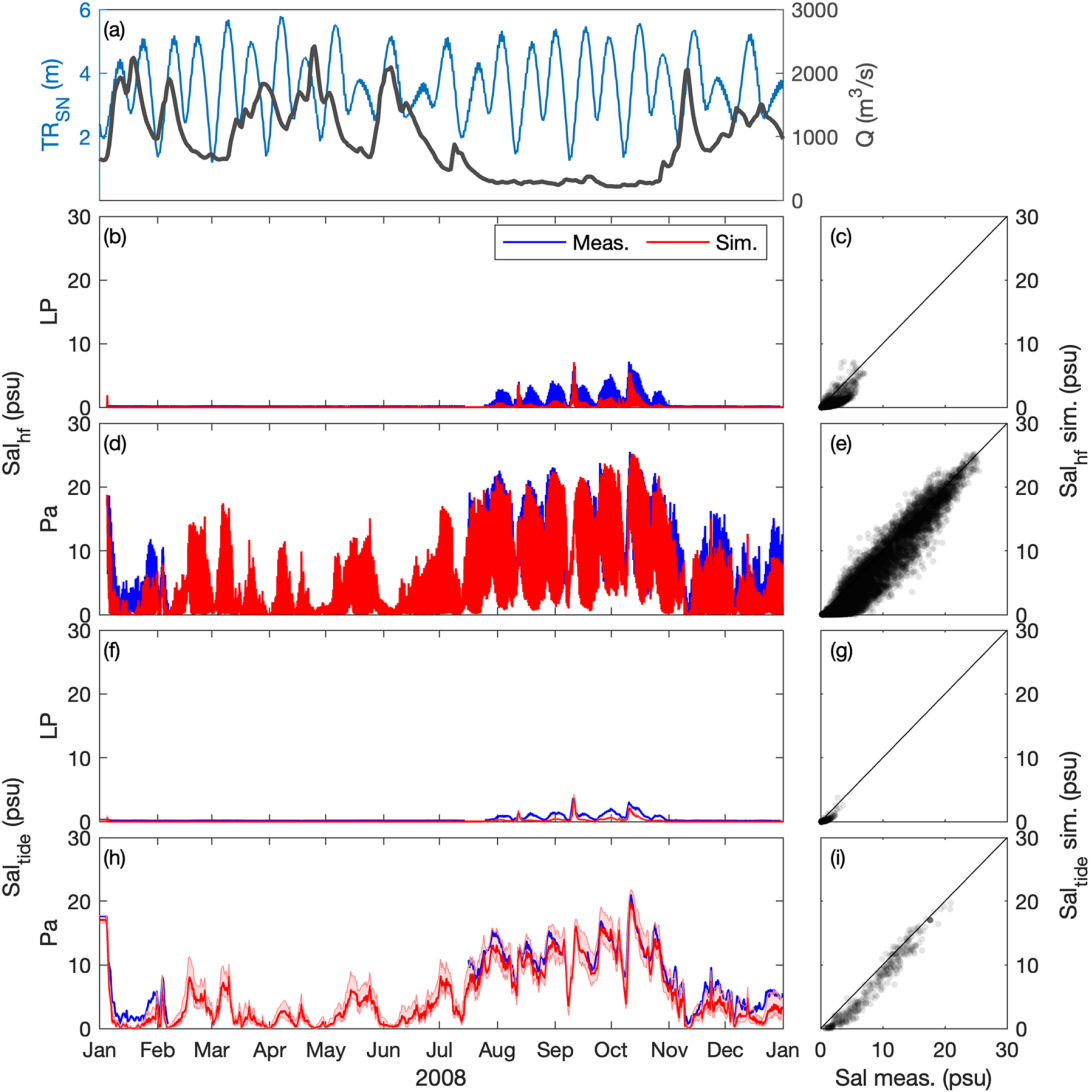
Hindcast validation of salinity *Sal* along the Loire Estuary. (**a**) Loire River discharge *Q* at the upstream model boundary and tidal range *TR*_*SN*_ at Saint Nazaire ‘SN’ (blue). Surface salinity at (**b,c,f,g**) Le Pellerin ‘LP’ and (**d,e,h,i**) Paimboeuf ‘Pa’ (see station locations in Fig. 1c). (Left panels) Measurements (blue) and simulations (red), and (right panels) simulations versus measurements, from January to December 2008. (**b**–**e**) High-frequency salinity *Sal*_*hf*_ (i.e., every 30 minutes) and (**f**–**i**) tide-averaged salinity *Sal*_*tide*_.

**Fig. 6:**
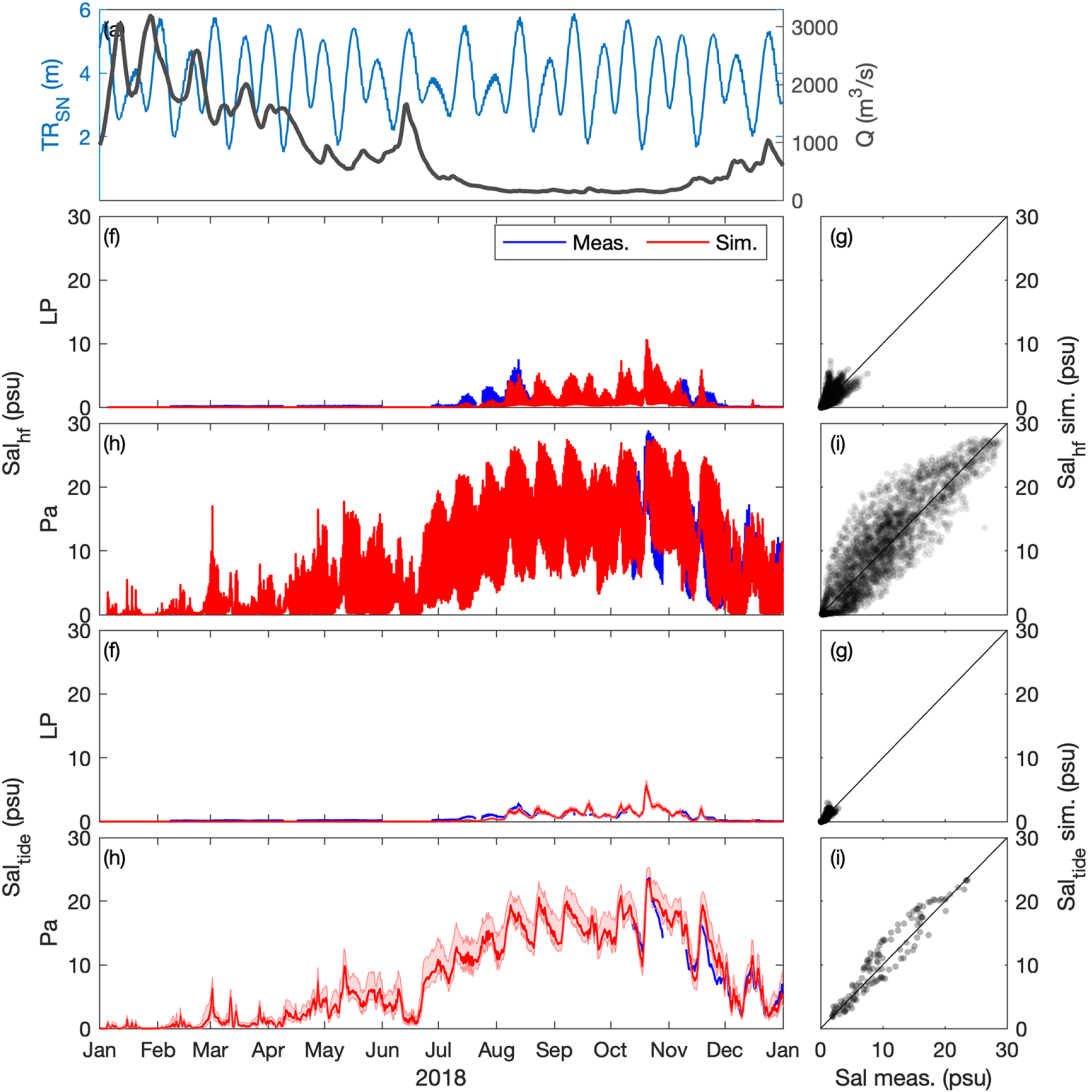
Hindcast validation of salinity *Sal* along the Loire Estuary. (**a**) Loire River discharge *Q* at the upstream model boundary and tidal range *TR*_*SN*_ at Saint Nazaire ‘SN’ (blue). Surface salinity at (**b,c,f,g**) Le Pellerin ‘LP’ and (**d,e,h,i**) Paimboeuf ‘Pa’ (see station locations in Fig. 1c). (Left panels) Measurements (blue) and simulations (red), and (right panels) simulations versus measurements, from January to December 2018. (**b**–**e**) High-frequency salinity *Sal*_*hf*_ (i.e., every 30 minutes) and (**f**–**i**) tide-averaged salinity *Sal*_*tide*_.

The surface salinity (at 1-m below the surface) is compared at Paimboeuf ‘Pa’ and Le Pellerin ‘LP’ stations (Fig. 1c), within the maximal salinity gradient area. High-frequency (i.e., every 30 minutes) salinity dynamics are well captured by the model along hydrological cycles (Figs. 5b–e, 6b–e). The model underestimates salinity at Le Pellerin in 2008, but the salinity intrusion during the low river discharge period (i.e., from August to November) is well reproduced. In Figs. 5f–I, 6f–i the tide-averaged salinity comparison further illustrates the model’s ability to simulate salinity variations at the hydrological and neap-spring time scales with good skills (*r*^2^ ≥ 0.7; Table 2).

### Suspended sediment concentration

**Fig. 7:**
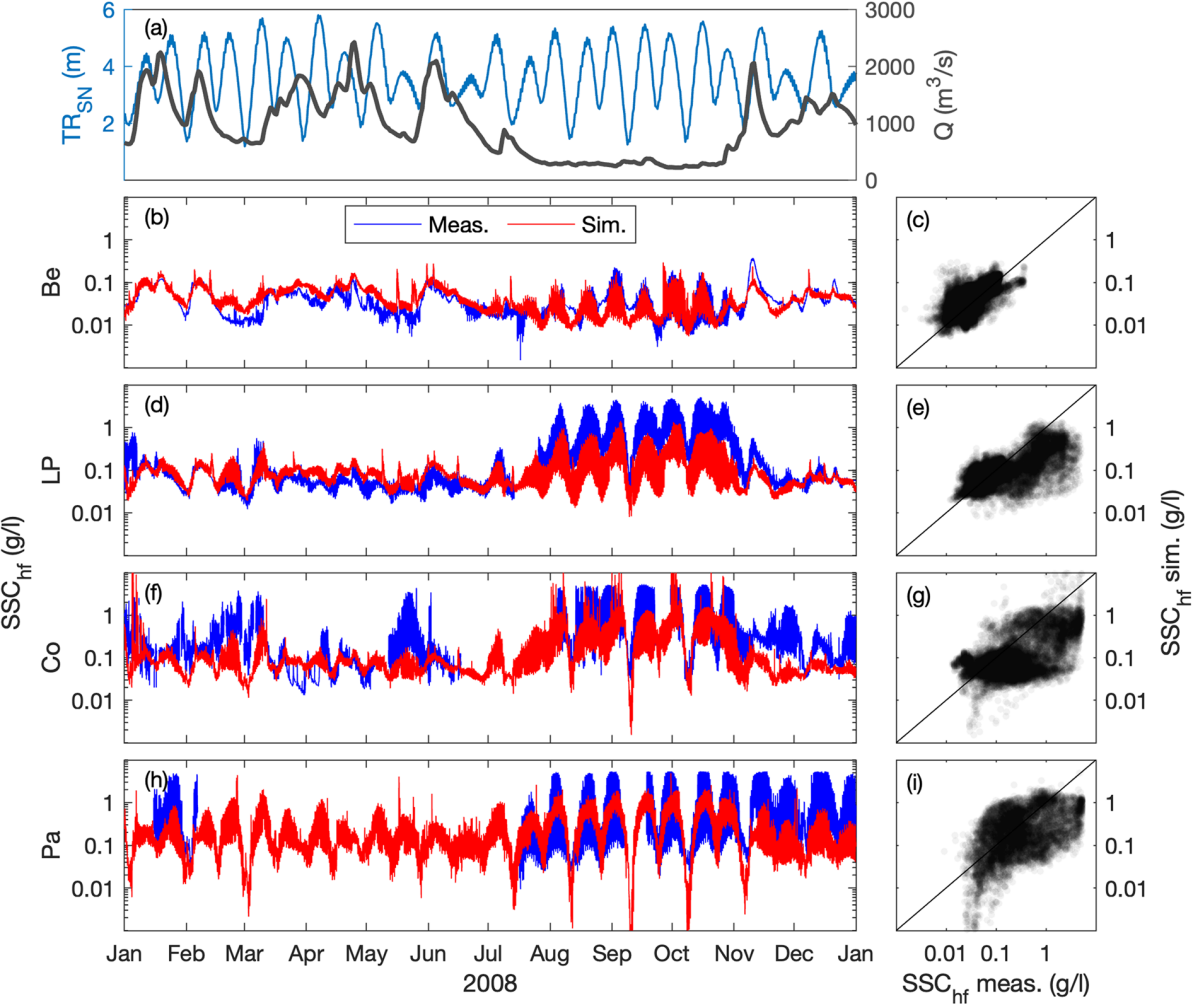
Hindcast validation of high-frequency suspended sediment concentration *SSC*_*hf*_ along the Loire Estuary. (**a**) Loire River discharge *Q* at the upstream model boundary and tidal range *TR*_*SN*_ at Saint Nazaire ‘SN’ (blue). Surface *SSC*_*hf*_ at (**b,c**) Bellevue ‘Be’, (**d,e**) Le Pellerin ‘LP’, (**f,g**) Cordemais ‘Co’, and (**h,i**) Paimboeuf ‘Pa’ (see station locations in Fig. 1c). (Left panels) Measurements (blue) and simulations (red), and (right panels) simulations versus measurements, from January to December 2008.

**Fig. 8:**
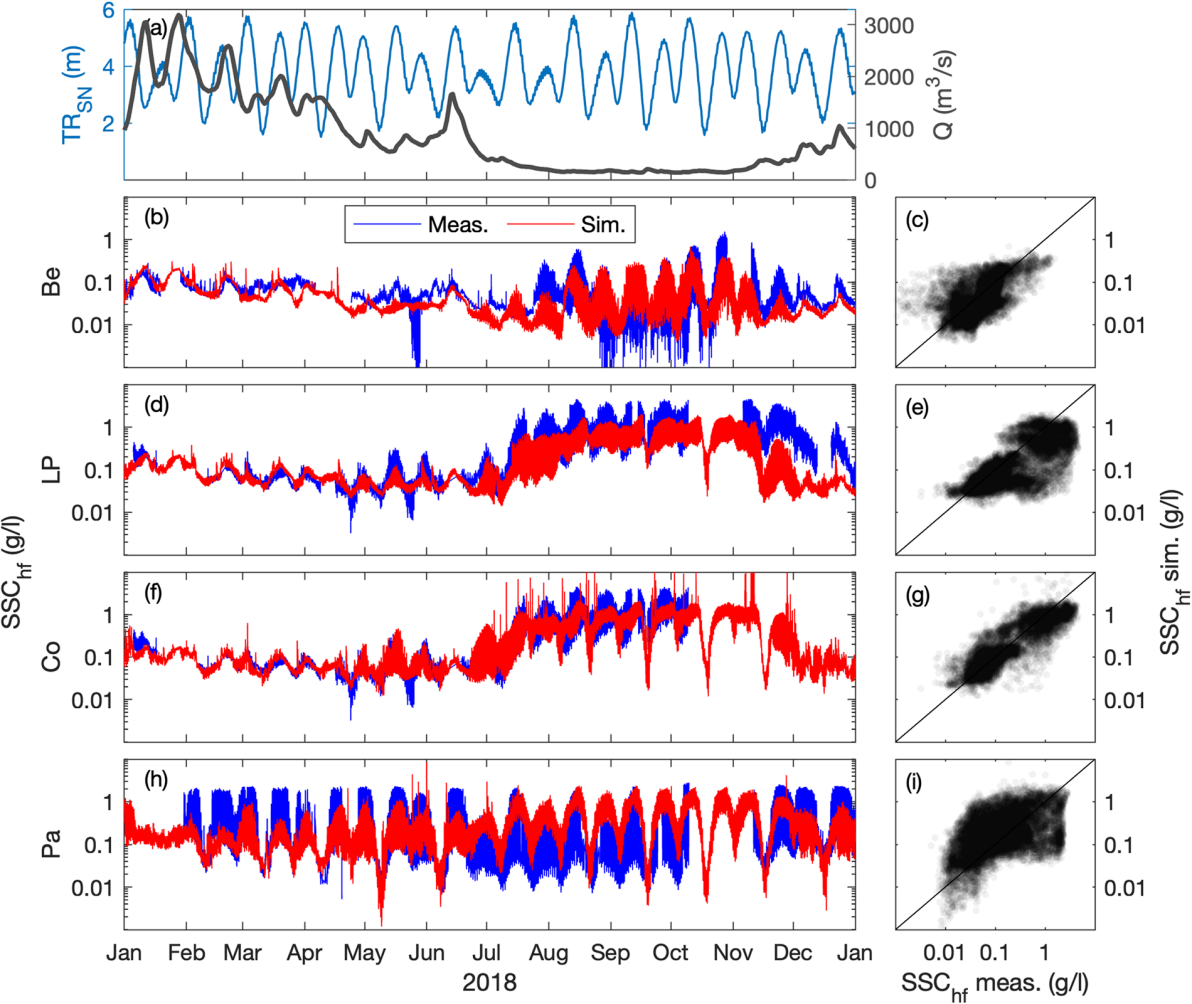
Hindcast validation of high-frequency suspended sediment concentration *SSC*_*hf*_ along the Loire Estuary. (**a**) Loire River discharge *Q* at the upstream model boundary and tidal range *TR*_*SN*_ at Saint Nazaire ‘SN’ (blue). Surface *SSC*_*hf*_ at (**b,c**) Bellevue ‘Be’, (**d,e**) Le Pellerin ‘LP’, (**f,g**) Cordemais ‘Co’, and (**h,i**) Paimboeuf ‘Pa’ (see station locations in Fig. 1c). (Left panels) Measurements (blue) and simulations (red), and (right panels) simulations versus measurements, from January to December 2018.

**Fig. 9:**
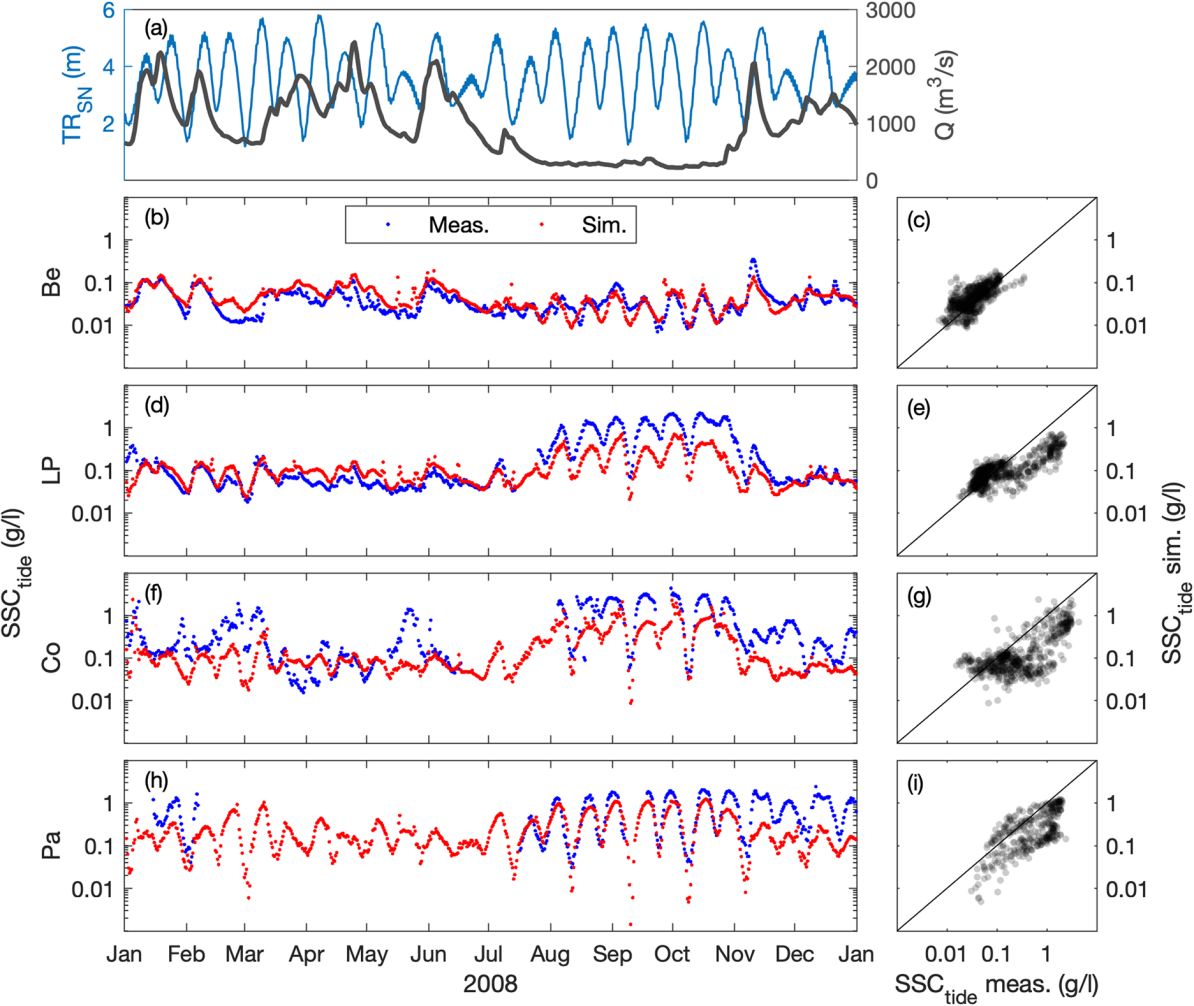
Hindcast validation of tide-averaged suspended sediment concentration *SSC*_*tide*_ along the Loire Estuary. (**a**) Loire River discharge *Q* at the upstream model boundary and tidal range *TR*_*SN*_ at Saint Nazaire ‘SN’ (blue). Surface *SSC*_*tide*_ at (**b,c**) Bellevue ‘Be’, (**d,e**) Le Pellerin ‘LP’, (**f,g**) Cordemais ‘Co’, and (**h,i**) Paimboeuf ‘Pa’ (see station locations in Fig. 1c). (Left panels) Measurements (blue) and simulations (red), and (right panels) simulations versus measurements, from January to December 2008.

**Fig. 10:**
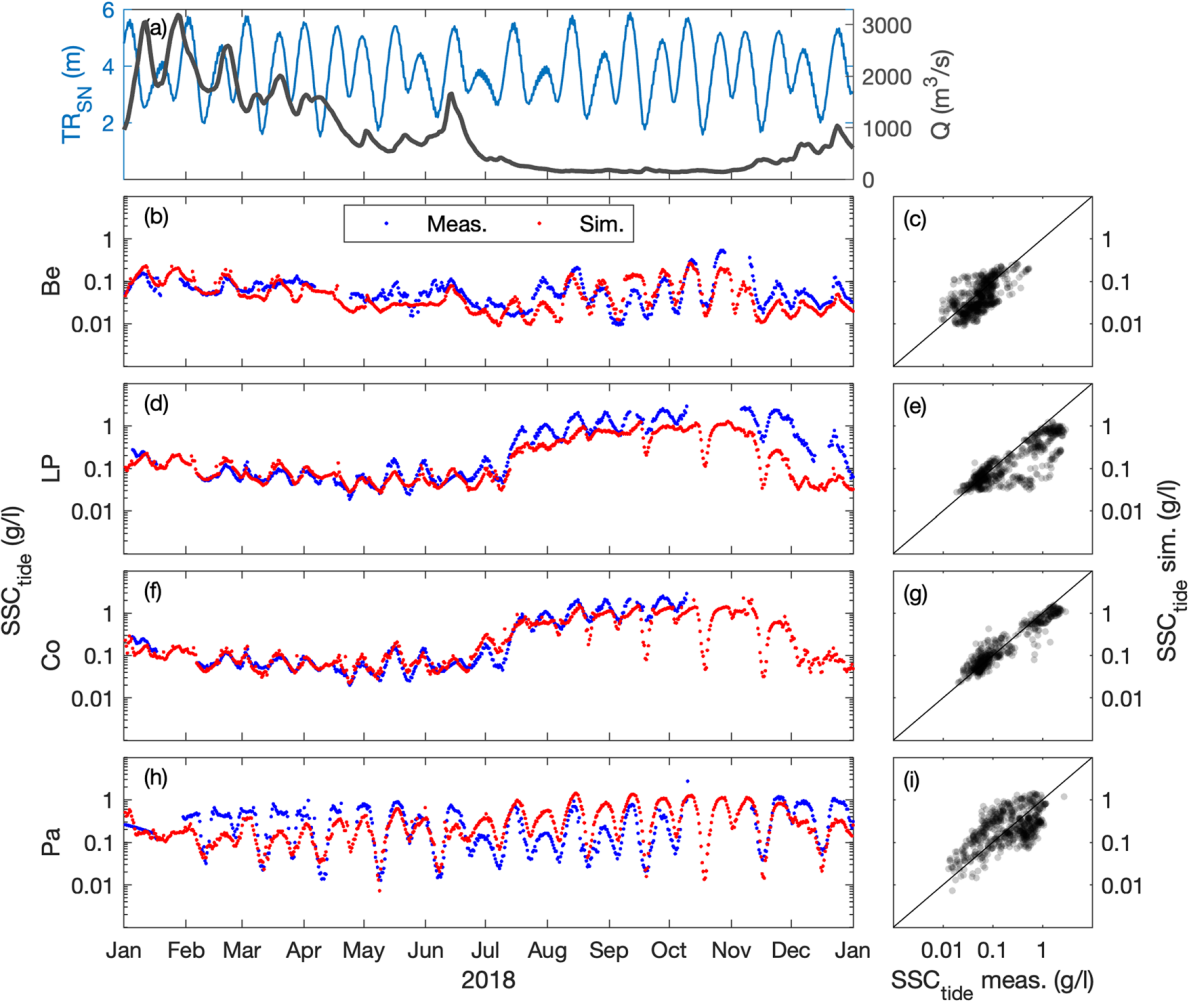
Hindcast validation of tide-averaged suspended sediment concentration *SSC*_*tide*_ along the Loire Estuary. (**a**) Loire River discharge *Q* at the upstream model boundary and tidal range *TR*_*SN*_ at Saint Nazaire ‘SN’ (blue). Surface *SSC*_*tide*_ at (**b,c**) Bellevue ‘Be’, (**d,e**) Le Pellerin ‘LP’, (**f,g**) Cordemais ‘Co’, and (**h,i**) Paimboeuf ‘Pa’ (see station locations in Fig. 1c). (Left panels) Measurements (blue) and simulations (red), and (right panels) simulations versus measurements, from January to December 2018.

**Fig. 11:**
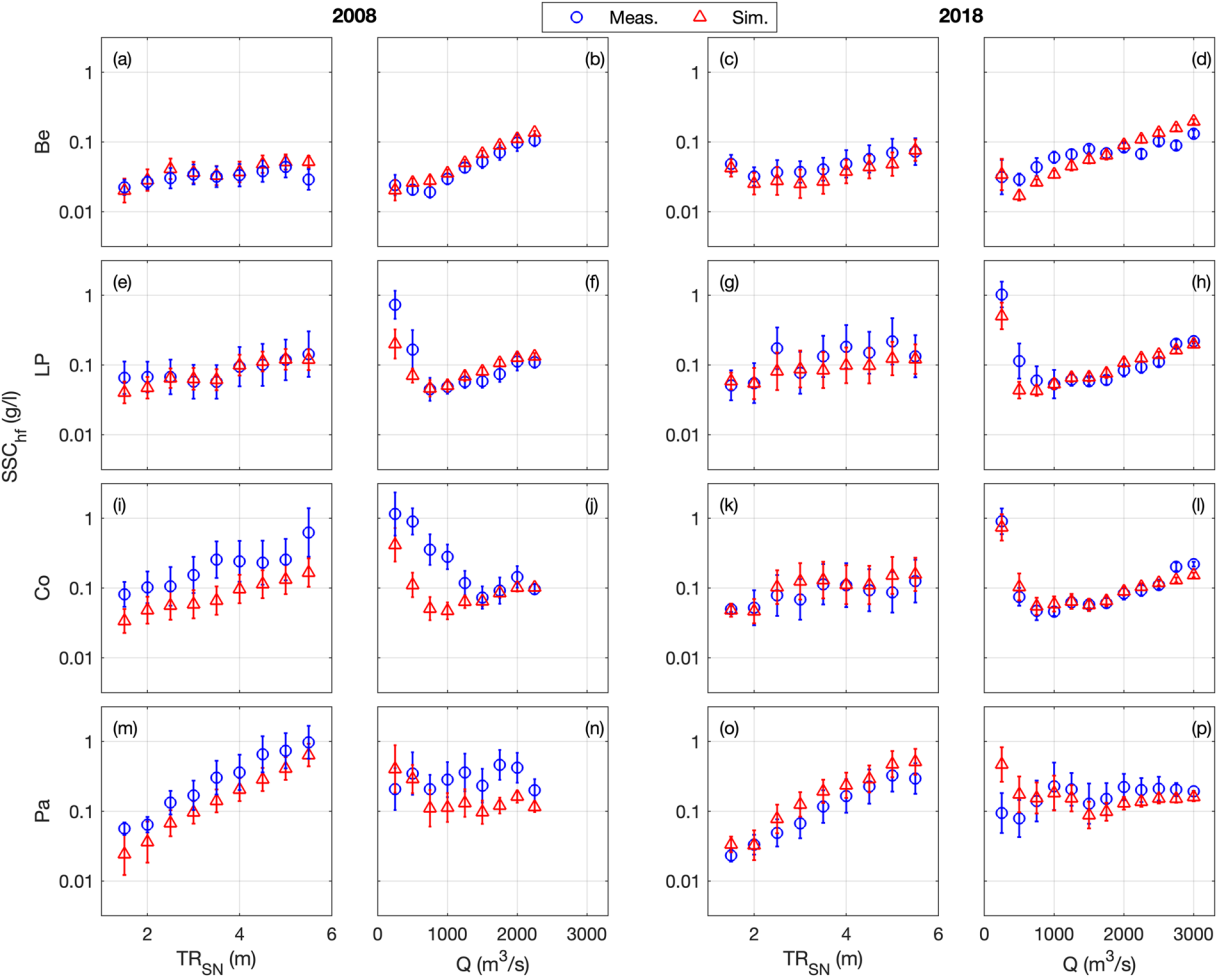
Hindcast validation of high-frequency suspended sediment concentration *SSC*_*hf*_ along the Loire Estuary versus the tidal range *TR*_*SN*_ at Saint Nazaire ‘SN’ and the Loire River discharge *Q* at the upstream model boundary. Surface *SSC*_*hf*_ at (**a**–**d**) Bellevue ‘Be’, (**e**–**h**) Le Pellerin ‘LP’, (**i**–**l**) Cordemais ‘Co’, and (**m**–**p**) Paimboeuf ‘Pa’ (see station locations in Fig. 1c). Measurements (blue circles) and simulations (red triangles) from January to December 2008 (two left panels) and from January to December 2018 (two right panels). Symbols represent class-averaged *SSC*_*hf*_ values (i.e., every 0.5 m for *TR*_*SN*_ and every 250 m^3^/s for *Q*), and brackets represent standard deviation.

The surface SSC (at 1-m below the surface) is compared at five stations along the estuary, where the turbidity maximum takes place (i.e., Paimboeuf ‘Pa’, Cordemais ‘Co’, Le Pellerin ‘LP’, and Bellevue ‘Be’; Fig. 1c). The seasonal and neap-spring variability of high-frequency SSC is reasonably well captured by the model at the different stations, but there is a main underestimation of highly-turbid events (Figs. 7, 8). This is especially visible at the downstream stations during summer (i.e., LP, Co, and Pa). Such underestimations are relatively common in the numerical modelling of estuarine sediment dynamics^53^, as monitoring stations along the shore may measure large and local sediment resuspensions that models cannot capture with a 50 to 100 m resolution^21^. However, the model presents better skills at tidal timescales (Table 2), providing confidence in its ability to simulate the main SSC levels along the year (Figs. 9, 10). This is confirmed by the comparison of measured and simulated SSC in function of tidal range and river discharge conditions (Fig. 11). We observe that the model underestimates the SSC at Cordemais and Le Pellerin in 2008 (Fig. 11i,j,m,n), which is not the case in the wetter year 2018 (Fig. 11k,l,o,p). In addition, the model proves to be able to simulate the main tidal and river dynamics.

The model validation of the Loire Estuary hydrodynamics, salinity, and sediment dynamics provides a sufficient level of confidence for using the numerical hindcast in various interdisciplinary studies. However, it is important to acknowledge the model’s limitations and errors (Table 2) to properly use the derived environmental parameters. For instance, this hindcast provides reasonable estimates of SSC changes in terms of order of magnitude, from tidal to hydrological timescales, but it is associated with greater uncertainties at sub-tidal timescales.

## Data Availability

The MARS3D-MUSTANG model chain used to provide the hindcast is an open-access software: https://mars3d.ifremer.fr. The source codes and parameter files are available on the CurviLoire Hindcast repository^51^.
